# Concurrent Disruption of Genetic Interference and Increase of Genetic Recombination Frequency in Hybrid Rice Using CRISPR/Cas9

**DOI:** 10.3389/fpls.2021.757152

**Published:** 2021-10-01

**Authors:** Chaolei Liu, Yiwei Cao, Yufeng Hua, Guijie Du, Qing Liu, Xin Wei, Tingting Sun, Jianrong Lin, Mingguo Wu, Zhukuan Cheng, Kejian Wang

**Affiliations:** ^1^ State Key Laboratory of Rice Biology, China National Rice Research Institute, Hangzhou, China; ^2^ State Key Laboratory of Plant Genomics and Center for Plant Gene Research, Institute of Genetics and Developmental Biology, Chinese Academy of Sciences, Beijing, China; ^3^ National Nanfan Research Institute (Sanya), Chinese Academy of Agricultural Sciences, Sanya, China

**Keywords:** genetic diversity, genetic interference, genetic recombination, *ZEP1*, synaptonemal complex, hybrid rice, genome editing

## Abstract

Manipulation of the distribution and frequency of meiotic recombination events to increase genetic diversity and disrupting genetic interference are long-standing goals in crop breeding. However, attenuation of genetic interference is usually accompanied by a reduction in recombination frequency and subsequent loss of plant fertility. In the present study, we generated null mutants of the *ZEP1* gene, which encodes the central component of the meiotic synaptonemal complex (SC), in a hybrid rice using CRISPR/Cas9. The null mutants exhibited absolute male sterility but maintained nearly unaffected female fertility. By pollinating the *zep1* null mutants with pollen from *indica* rice variety 93-11, we successfully conducted genetic analysis and found that genetic recombination frequency was greatly increased and genetic interference was completely eliminated in the absence of *ZEP1*. The findings provided direct evidence to support the controversial hypothesis that SC is involved in mediating interference. Additionally, the remained female fertility of the null mutants makes it possible to break linkage drag. Our study provides a potential approach to increase genetic diversity and fully eliminate genetic interference in rice breeding.

## Introduction

Plant breeding aims to develop superior varieties to suit the needs of farmers and consumers (Moose and Mumm, 2008). However, innovations in breeding materials strongly depend on creating novel allele combinations that bring together advantageous alleles and remove linked, disadvantageous alleles. This is traditionally limited by the number of crossovers (COs) during meiosis (Taagen et al., 2020). To generate sufficient genetic diversity, breeders and geneticists are exploring approaches to increase the CO frequency, alter CO distribution, or induce COs between non-homologous chromosomal regions (Mieulet et al., 2018; Blary and Jenczewski, 2019).

The synaptonemal complex (SC) is a meiosis-specific structure involved in CO formation and chromosome segregation (Gao and Colaiácovo, 2018). SC assembly starts from early prophase I and forms a tripartite structure at pachytene, which consists of axial or lateral elements, transverse filaments, and central element (Moses, 1969; de Boer and Heyting, 2006; Fraune et al., 2016). SC dynamics are tightly regulated by its components for the proper SC assembly (Gao and Colaiácovo, 2018). Among them, TF proteins locate at the central of SC and act important roles in bridging the parallel homologous axes (Dubois et al., 2019). Usually, the N-terminal domain of the TF protein is positioned in the middle of the SC, while its C terminus is located next to the lateral elements (Anderson et al., 2005; Schild-Prüfert et al., 2011). Such special organization pattern is critical for the function of the SC. The first reported TF protein gene is *ZIP1*, which was identified in budding yeast (Sym et al., 1993; Storlazzi et al., 1996). *zip1* mutants exhibit defects in SC formation and genetic recombination; however, its homologous genes in other organisms appear to play opposite roles on CO frequency. *Sycp1* encodes a TF protein in mouse, and *Sycp1^−/−^* mice shows 90% of COs disappeared (de Vries et al., 2005). Mutation of *c*(3)*G*, the TF protein gene in *Drosophila*, causes the loss of all COs (Page and Hawley, 2001). Unexpectedly, the *syp-1* mutants in the roundworm *Caenorhabditis elegans* exhibit a severe reduction in COs (MacQueen et al., 2002), while knockdown of *SYP-1* by RNA interference (*RNAi*) increases the CO frequency (Libuda et al., 2013). The *ZIP1* orthologs have also been identified in plant species including *Arabidopsis thaliana*, rice (*Oryza sativa*), maize (*Zea mays*), wheat (*Triticum aestivum*), and barley (*Hordeum vulgare*; Higgins et al., 2005; Wang et al., 2010; Golubovskaya et al., 2011; Khoo et al., 2012; Barakate et al., 2014). Suppression of *ZYP1* in *Arabidopsis thaliana* or in barley (*H. vulgare*) led to reduced CO frequency (Higgins et al., 2005; Barakate et al., 2014). In contrast with *Arabidopsis* and barley, the *Tos17* insertion mutant lines of *ZEP1* in rice had more COs than the wild type (WT; Wang et al., 2010; Wang K. et al., 2015).

Genetic interference, a phenomenon where the occurrence of one crossover (CO) inhibits the formation of other COs nearby on the same chromosome pair, imparts a remarkable level of regulation of the number and distribution of COs per chromosome in eukaryotes (Sun et al., 2017; Otto and Payseur, 2019). Therefore, reduction or disruption of genetic interference can be considered to increase genetic diversity in crop breeding (Taagen et al., 2020). Presently, a number of studies have revealed that the strength of genetic interference increased as the extent of synapsis increased (Libuda et al., 2013; Zhang et al., 2014; Wang K. et al., 2015). However, in some organisms, evidence indicates that genetic interference is exerted prior to the SC assembly (Bishop and Zickler, 2004; Fung et al., 2004). Partly because of the lack of null TF mutants, the exact role of SC in CO interference is still enigmatic. Most recently, the null mutants of *zyp1* have been generated in *Arabidopsis thaliana*, and cytological and genetic analysis reveals that ZYP1 is required for CO interference (Capilla-Pérez et al., 2021; France et al., 2021). However, researches of null mutants of TF protein genes in cereal crops are scarce to date.

In the present study, we selected the inter-subspecific hybrid rice variety Chunyou84 (CY84), an elite inter-subspecific hybrid rice from a cross between the maternal Chunjiang 16 A (16 A), a *japonica* male-sterile line, and the paternal C84, an *indica*-*japonica* intermediate-type line, as the acceptor and used the CRISPR/Cas9 technology to generate *zep1* null mutants. By genetic analysis, we revealed that genetic interference is completely eliminated in the absence of *ZEP1*, indicating an important role of SC in mediating interference in rice. In addition, the way of generating *zep1* null mutants with increased genetic recombination frequency and eliminated genetic interference is a promising approach to increase genetic diversity in rice breeding.

## Materials and Methods

### Vector Construction

The CRISPR-Cas9 vector for complete knockout of *ZEP1* was constructed by the isocaudomer ligation method, as described in Wang C. et al. (2015). The annealed *ZEP1* g++/*ZEP1* g-- oligonucleotides (Supplementary Table 1) were ligated into the SK-sgRNA vector that digested with *AarI*. Then, the sgRNA of *ZEP1* (digested with *KpnI* and *BglII*) was assembled into the pC1300-*Actin*:Cas9 binary vector (digested with *KpnI* and *BamHI*) to obtain the vector pC1300-*Actin*:Cas9-sgRNA*^ZEP1^* for generation of *zep1* null mutants.

### Rice Transformation and Growth Conditions

The binary vector pC1300-*Actin*:Cas9-sgRNA*^ZEP1^* was introduced into *Agrobacterium tumefaciens* (Strain EHA105). The *Agrobacterium* was then transformed into calluses derived from CY84 to generate transgenic lines. Fifteen independent transgenic plants of pC1300-*Actin*:Cas9-sgRNA*^ZEP1^* were obtained and grown in summer in the transgenic paddy fields of the China National Rice Research Institute in Hangzhou, China.

### Mutant Screening

Genomic DNA of transgenic plants was extracted from approximately 50mg fresh leaf tissue *via* the cetyltrimethylammonium bromide (CTAB) method. PCR was performed with KOD FX DNA polymerase (Toyobo, Japan) to amplify the fragments surrounding *ZEP1* target site. Primers *ZEP1* F and *ZEP1* R are listed in Supplementary Table 1. The mutants were detected according to the published Hi-TOM procedure (Liu et al., 2019).

### Cytological Analyses

Panicles at meiosis stage were harvested and fixed in Carnoy’s solution (ethanol:glacial acetic acid, 3:1) for more than 24h at room temperature. Microsporocytes were squashed on a slide with a needle and covered with a coverslip. Then, slides were frozen in liquid nitrogen, and the coverslips were removed rapidly with a blade. Chromosomes were stained with 4′,6-diamidino-2-phenylindole (DAPI) in an antifade solution (Vector Laboratories, Burlingame, CA, United States) as described in (Wang et al., 2009). Fluorescence microscopy was conducted using an Olympus BX61 fluorescence microscope fitted with a micro charge-coupled device camera.

### Fluorescence Immunolocalization

Fresh young rice panicles were fixed in 4% (w/v) paraformaldehyde for 45min at room temperature. Anthers at proper meiosis stage were squashed with a needle in PBS solution and covered with a coverslip. After soaking in liquid nitrogen and removing the coverslip, slides were then incubated in a humid chamber at 37°C for 4h with anti-REC8 (Mouse) and anti-ZEP1 (rabbit) polyclonal antibodies (diluted 1:500 in TNB buffer: 0.1M Tris–HCl, pH 7.5, 0.15M NaCl, and 0.5% blocking reagent). After three rounds of washing in PBS, Texas red-conjugated goat anti-mouse antibody and fluorescein isothiocyanate-conjugated sheep anti-rabbit antibody (1:1,000) were added to the slides. The chromosomes were counterstained with DAPI in an antifade solution (Vector Laboratories, Burlingame, CA, United States). Fluorescence microscopy was performed as described above.

### Genotyping *via* Hi-TOM

Single nucleotide polymorphisms (SNPs) markers were developed on the basis of the whole-genome sequences of C84 and chunjiang16A (Wang et al., 2019). The primers are listed in Supplementary Table 1. The Hi-TOM method was performed for the genotyping as previously described in (Liu et al., 2019).

### Interference Strength Analysis

The analysis of interference strength was described in Wang K. et al. (2015). For each pair of adjacent intervals, the coefficient of coincidence analysis compared the observed frequency of CO occurring in both intervals with the frequency expected if the CO occurred independently in the two intervals, with the interference strength calculated as (1-observed/expected). The expected number of chromosomes with CO occurring in both intervals (XY and YZ) was calculated as: (recombination frequency between X and Y)×(recombination frequency between Y and Z)×(number of chromosomes examined). See the details in Supplementary Tables 2 and 3.

### Genetic Recombination Analysis

The WT (CY84) and *zep1-KO* plants were crossed with the *indica* variety 93-11 to generate the segregation population. The hybrid seeds were soaked in deionized water at 37°C in the dark for 2days and then transferred to a net floating on deionized water for further 5days. The seedlings were cultured in a half-strength Kimura B nutrient solution (pH 5.4; Liu et al., 2020). The genotypes of each plant in the segregation population were identified by Hi-TOM. The genotypes of each female gamete of CY84 and *zep1-KO* plants were deduced and listed in Supplementary Tables 4–7. Then, they were used for the frequency and distribution of genetic recombination analysis. For each pair of adjacent markers (X and Y), the recombination frequency was calculated as: (number of chromosomes with one CO between X and Y/number of chromosomes examined).

## Results

### Selection of Gene-Editing Target Site and Acquisition of *ZEP1* Mutants

**Figure 1:**
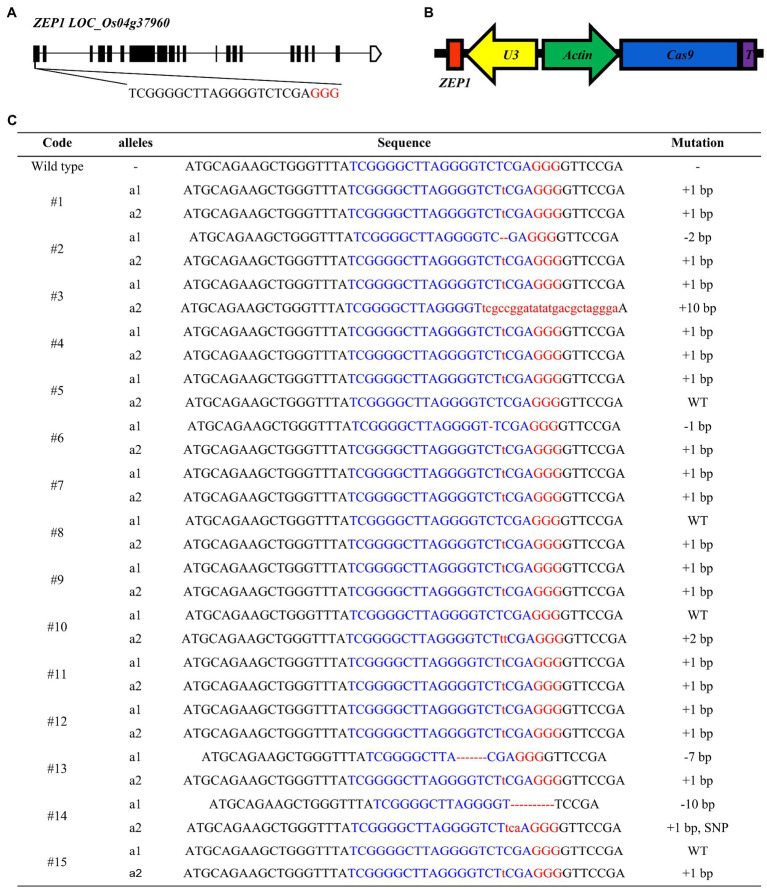
Genome editing of *ZEP1* in hybrid rice. **(A)** *ZEP1* gene structure and gRNA target site. **(B)** The structure of CRISPR-Cas9 vector targeting *ZEP1*. **(C)** Mutations in *ZEP1* editing lines in the Chunyou84 (CY84) background. The protospacer adjacent motif sequence is highlighted in red. Red lowercase letters and dashed lines indicate inserted or deleted nucleotides, respectively.

*ZEP1* has a full length of 9.478-kbp, distributed in 21 exons and 20 introns and encodes a transverse filament protein containing 869 amino acids (Figure 1A). ZEP1 comprises coiled-coil proteins with globular domains at its N termini from amino acids 1 to 63 and C termini from amino acids 714 to 869. The C-terminal globular domain of 156 residues putatively binds DNA, while the N-terminal globular domain of 63 residues is basic (Wang et al., 2010). The *Tos17* insertion mutant lines of *ZEP1* used in previous studies are partial loss-of-function mutants (Wang et al., 2010; Wang K. et al., 2015), which still contain the N-terminal residues. To generate *zep1* knockout mutants, we employed the clustered regularly interspaced short palindromic repeats (CRISPR)/CRISPR-associated protein 9 (Cas9) system to edit *ZEP1* and chose the 19th to the 38th nucleotides in the first exon near the initiation codon as our target site (Figure 1A). We successfully constructed the CRISPR-Cas9 vector pC1300-*Actin*:Cas9-sgRNA*^ZEP1^* (Figure 1B) and then introduced it into the elite inter-subspecific hybrid rice variety Chunyou84 (CY84) *via Agrobacterium*. A total of fifteen independent transgenic plants were obtained in the T_0_ generation with six homozygous mutations (#1, #4, #7, #9, #11, and #12) and five biallelic mutations (#2, #3, #6, #13, and #14; Figure 1C).

### Null Mutants of *ZEP1* in Rice

**Figure 2:**
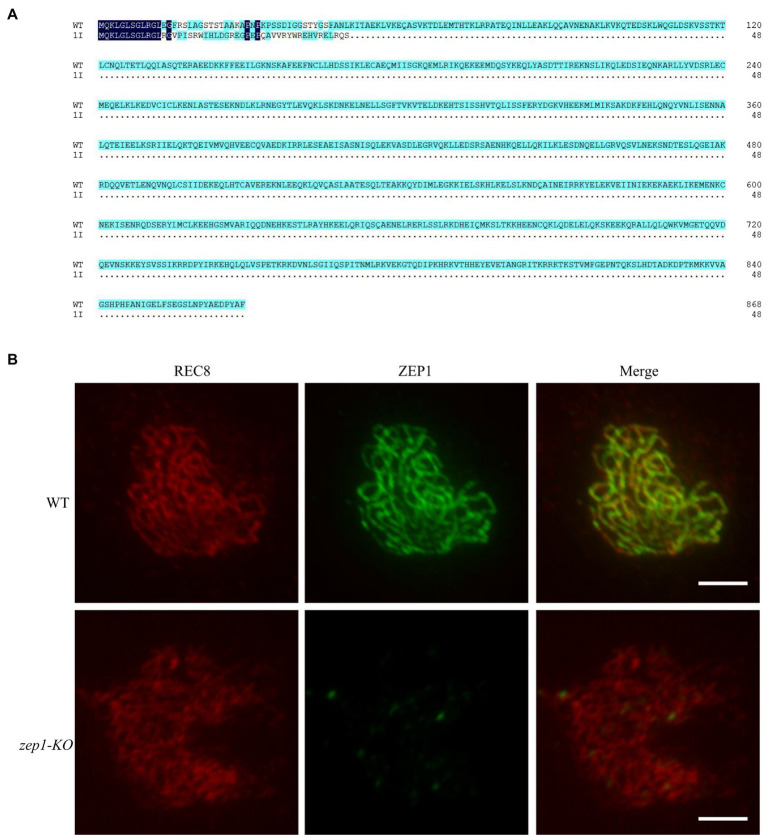
Generation of *zep1* null mutants. **(A)** Amino acid sequence alignment of ZEP1 protein of WT and mutants. The *ZEP1* gene encodes a transverse filament protein containing 869 amino acids, while 1bp insertion caused the premature transcription termination of *ZEP1*. **(B)** Immunostaining of REC8 (red) and ZEP1 (green) at pachytene stage in the WT and *zep1-KO* plants. Bar=5μm. REC8 was used to indicate the meiotic chromosomes.

To clearly uncover the editing mutations of *ZEP1* on genetic recombination frequency and genetic interference, we selected the 1-bp insertion homozygous mutants for further researches. As shown in Figure 2A, the 1-bp insertion caused premature termination of *ZEP1* transcription and ultimately encoded a severely truncated protein containing 48 residues, of which only 12 residues were consistent with that of WT. Because of the basic of ZEP1 N-terminal globular domain, we speculated the 1-bp insertion mutants are *ZEP1* knockout lines, which differ from the previous partial loss-of-function *zep1* mutants (Wang et al., 2010; Wang K. et al., 2015). To confirm the absolute absence of ZEP1, we conducted immunostaining by using anti-REC8 and anti-ZEP1 antibodies. REC8 is a component of the cohesion complex and is required for sister chromatid cohesion, axial element formation, and homolog pairing and is used as a marker to monitor early meiotic events during prophase I here (Zhang et al., 2006). In the WT plants, ZEP1 signals were robust at pachytene, while in the mutants, no signals except the background were detected in any observed meiocytes (*n*=500; Figure 2B). These results implied that *ZEP1* was completely knocked out in the mutants. Hereafter, we renamed these mutants as *zep1-KO*.

### Effects of *ZEP1* Null Mutation on Sterility

**Figure 3:**
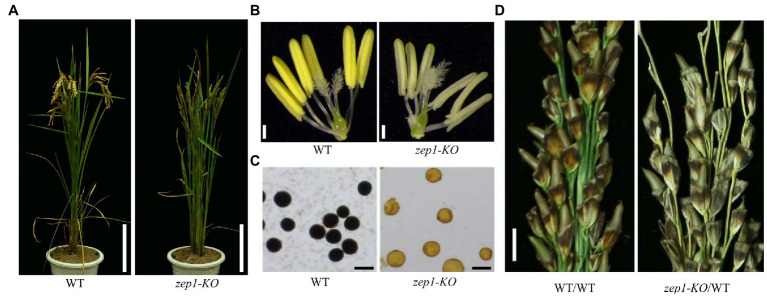
Effects of *ZEP1* knockout mutation on sterility. **(A)** Comparison of WT and *zep1-KO* plants at maturity. Bar=20cm. **(B)** Comparison of anthers and pistils of WT and *zep1-KO* plants at heading date. Bar=1mm. **(C)** Iodine-iodide kalium staining of pollen from the WT and *zep1-KO* plants. Bar=5μm (pollen). **(D)** Cross the WT and *zep1-KO* plants (as maternal plant) with pollen from WT plants. Bar=0.5cm.

Fertility is an important agronomic trait for genetic recombination analysis and plant breeding (Wang K. et al., 2015). There were no discernible differences in plant morphology between the WT and the *zep1-KO* lines (Figure 3A), but the anthers of *zep1-KO* plants were light white while those of the WT were bright yellow (Figure 3B). We further confirmed the suspected male sterility by 1% I_2_-KI solution staining and observed no staining in the pollens from the *zep1-KO* plants (Figure 3C). Therefore, unlike the previously described partial fertility of the *zep1* mutants (Wang et al., 2010; Wang K. et al., 2015), complete knockout of *ZEP1* caused absolute male sterility. In contrast to the anthers, the *zep1-KO* pistils seemed unaffected, similar to those of WT plants (Figure 3B). To determine whether the female gametes were fertile, we pollinated the *zep1-KO* and the control WT plants with pollen from WT plants. Both the WT and the *zep1-KO* plants could set seeds (Figure 3D), with a seed-setting rate of 38.1±5.8% and 34.4±8.5% for the WT and *zep1-KO*, respectively. These results suggested that the female meiocytes of *zep1-KO* mutants were not severely affected.

**Figure 4:**
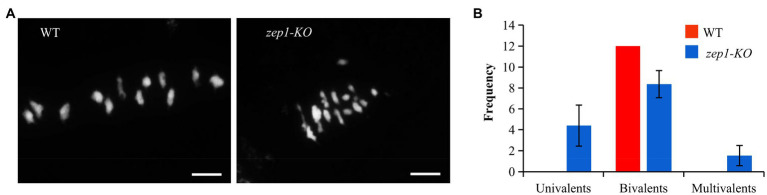
Chromosome behaviors of male meiocytes in WT and *zep1-KO* plants. **(A)** DAPI staining of the meiotic chromosomes of pollen mother cells (PMCs) from the WT and *zep1-KO* plants. Bar=5μm. **(B)** Frequency distribution of univalents, bivalents, and multivalents in the pollen mother cells (PMCs) from WT and *zep1-KO* plants. (*n*=30).

The complete male sterility in the *zep1-KO* plants might result from defects in meiosis. To test this hypothesis, we investigated the chromosome behaviors of male meiocytes by DAPI staining. In WT plants, all 12 bivalents in the pollen mother cells (PMCs) lined up on the equatorial plate at metaphase I (Figure 4A). However, univalents, bivalents, and multivalents were observed in the *zep1-KO* PMCs, numbering 4.40±1.96, 8.37±1.30, and 1.53±0.97 (*n*=30), respectively (Figure 4). Therefore, the presence of univalents and multivalents likely caused the male sterility of the *zep1-KO* lines.

### Effects of *ZEP1* Null Mutation on CO Formation

**Figure 5:**
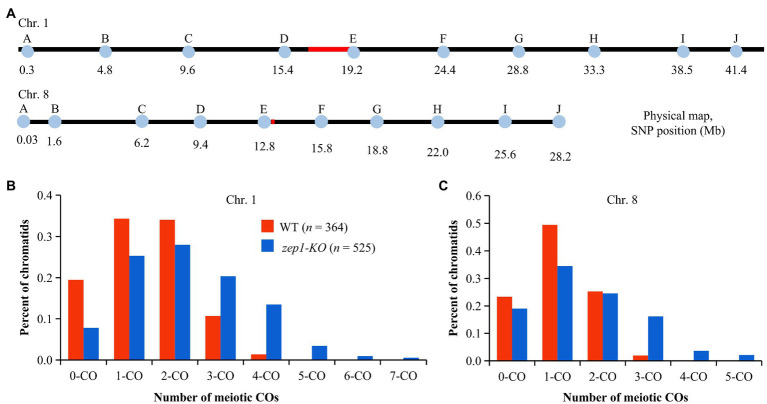
Analysis of chromatids in WT plants and *zep1* null mutants. **(A)** Physical positions of the SNP markers along chromosomes 1 and 8. **(B)** Percentage of total chromatids (*n*) for different types of COs on chromosome 1. **(C)** Percentage of total chromatids (*n*) for different types of COs on chromosome 8.

To explore whether the female recombination frequency was affected by disruption of *ZEP1*, we firstly crossed the WT (the CY84 hybrid) and *zep1-KO* lines with the *indica* rice variety 93-11 and then examined the genetic recombination events of WT×93-11 and *zep1-KO* × 93-11 F_1_ individuals *via* genotyping. To this end, we developed 10 nearly uniformly distributed single nucleotide polymorphism (SNP) markers along each of chromosomes 1 and 8 (Figure 5A) that were polymorphic between the parental lines of the CY84 hybrid. Then, we genotyped 364 individual F_1_ progeny of WT×93-11 and 525 individual F_1_ progeny of *zep1-KO* × 93-11 using those markers. After this, we tracked the recombination events that had occurred in the WT or *zep1-KO* parental plants (CY84 hybrid background) based on a previous method (Wang K. et al., 2015), using our SNP markers to deduce the genotype of each chromatid following meiosis and thus infer the number of COs that had occurred to produce each chromatid. In the WT plants, chromosome 1 chromatids with zero, single, double, triple, and quadruple COs occurred at frequencies of 20, 34, 34, 11, and 1%, respectively. By contrast, the *zep1-KO* mutants exhibited a decreased frequency of zero (8%), single (25%), and double (28%) COs but a higher frequency of triple (20%), quadruple (14%), quintuple (3%), sextuple (1%), and septuple (1%) COs (Figure 5B). Similar results were detected on chromosome 8, with 19% zero, 34% single 25% double, 16% triple, 4% quadruple, and 2% quintuple COs occurred in *zep1-KO* mutants, while the frequencies of zero, single, double, and triple COs occurred in WT plants were 23, 49, 25, and 2%, respectively (Figure 5C).

**Figure 6:**
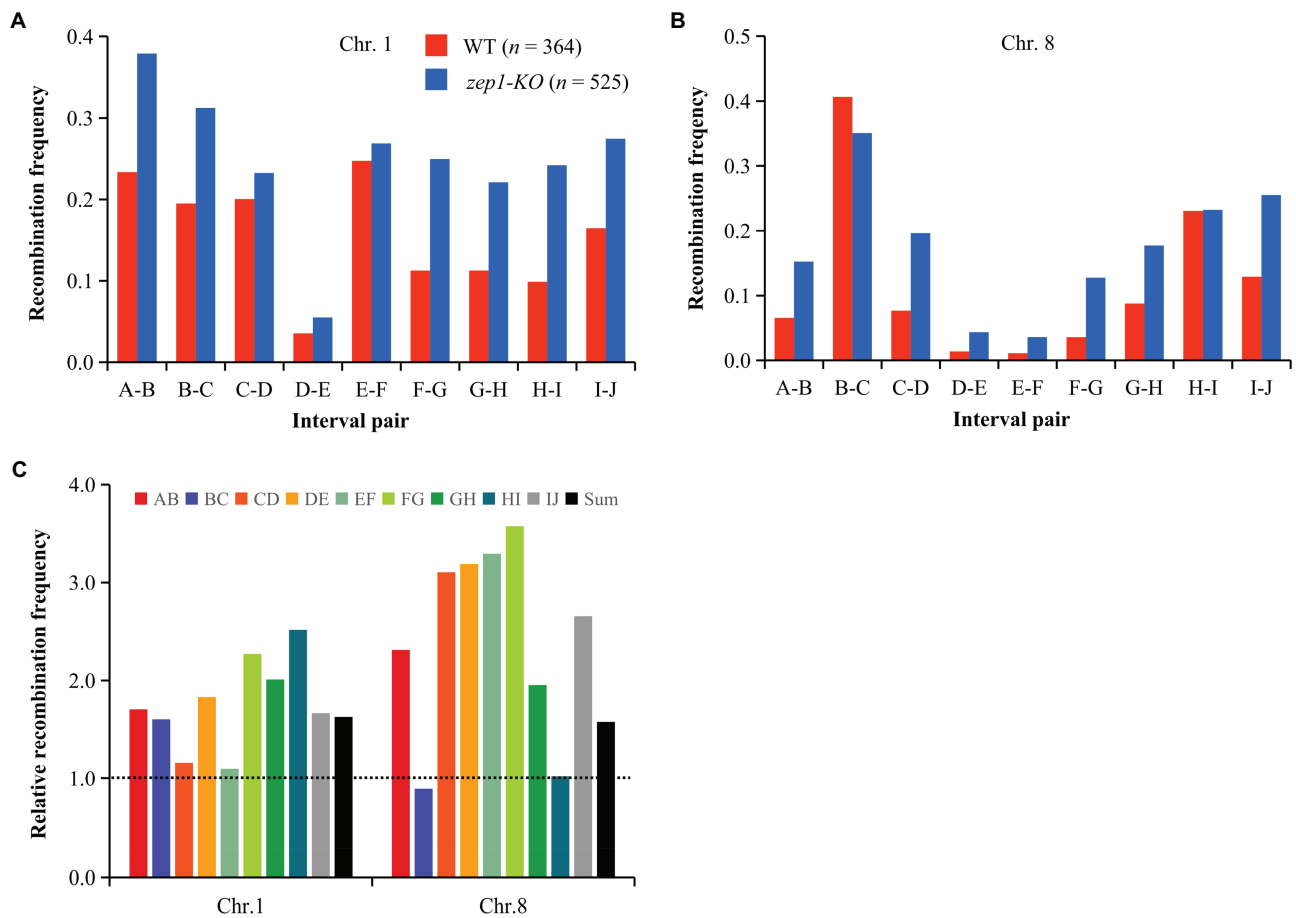
*zep1* null mutation increases recombination frequencies. **(A)** Recombination frequencies measured between adjacent SNP markers on chromosomes 1. **(B)** Recombination frequencies measured between adjacent SNP markers on chromosomes 8. **(C)** Relative recombination frequency (*zep1-KO*/WT) measured between adjacent SNP markers on the chromosomes 1 and 8.

We further calculated the recombination frequency of *zep1-KO* and WT plants. The average number of COs per chromatid on chromosomes 1 and 8 in WT plants was 1.40 and 1.06, respectively, while that of *zep1-KO* mutants was significantly higher, with 2.33 and 1.57 COs per chromatid on chromosomes 1 and 8, respectively (Figure 6). The recombination frequency was elevated in all identified intervals in the *zep1-KO* lines compared to that of the WT, varying from 1.01- to 3.57-fold (Figure 6). In particular, the recombination frequency of the DE interval on chromosome 1 and the EF interval on chromosome 8, both of which span the centromere, was elevated by 1.83- and 3.29-fold, which was higher than the average recombination frequency of entire chromosomes: 1.63- and 1.58-fold for chromosomes 1 and 8, respectively (Figure 6).

### Effects of *ZEP1* Null Mutation on Genetic Interference

**Figure 7:**
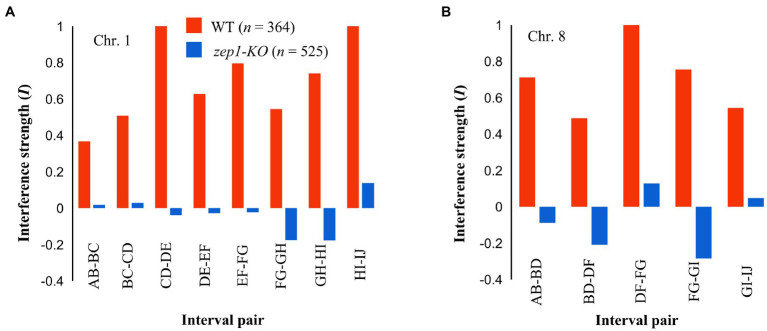
*zep1* null mutation disrupts crossover (CO) interference. Interference strength (*I*) values for different adjacent interval pairs of chromosomes 1 **(A)** and 8 **(B)** in WT and *zep1-KO* plants.

To assess whether genetic interference was affected by the null mutation of the *ZEP1* gene, we conducted coefficient of coincidence analysis according to the method described for *C. elegans* (Libuda et al., 2013). In the analysis, the strength of interference (*I*) equal to 1 is indicative of complete interference, while an *I* equal to zero is indicative of an absence of interference (Fernandes et al., 2018). In WT plants, the strength of interference (*I*) in eight adjacent interval pairs of chromosome 1 ranged from 0.37 to 1.00, indicating the existence of strong genetic interference between neighboring COs. By contrast, the *zep1-KO* mutants showed reduced *I* for those interval pairs, with *I* values of −0.18 to 0.14 (Figure 7A). For chromosome 8, the *I* value of the four measured interval pairs in the WT ranged from 0.48 to 0.71, whereas that in *zep1-KO* lines ranged from −0.17 to 0.05 (Figure 7B). These results suggest that genetic interference is likely fully eliminated in *zep1-KO* plants.

## Discussion

ZEP1 is the central element protein of the SC that regulates genetic recombination frequency in rice (Wang et al., 2010). The partial loss of function of this gene can increase genetic recombination frequency and attenuate CO interference (Wang K. et al., 2015), but the effects of *ZEP1* null mutations on CO formation and interference have not been reported comprehensively yet. The N-terminal domain of the ZEP1 is located in the middle of the SC, and this special organization pattern is critical for SC assembly (Anderson et al., 2005; Wang et al., 2010). In this study, we successfully generated null mutation of *ZEP1* in hybrid rice by genome editing. Genetic analysis of the null mutants revealed that genetic recombination frequency was greatly increased, and CO interference was completely eliminated in the absence of *ZEP1* in CY84 variety background. Compared with the partial loss of function of *ZEP1* mutants (Wang et al., 2010; Wang K. et al., 2015), the new null allele in this study showed stronger phenotype than those of previous weak alleles, indicating the residuary N-terminal domain of the ZEP1 in previous weak alleles might still play some roles in meiosis, although the SC cannot be assembled in the weak mutant.

Interference is a phenomenon that reduces the likelihood of a CO occurring adjacent to another CO along regions of a chromosome (Taagen et al., 2020). However, the relationship between the SC and genetic interference has long been a controversy in organisms. In budding yeast, the data of synapsis initiation complex (*SIC*) are assembled prior to SC and their proper position in the absence of SC formation demonstrated an aspect of interference that is independent of synapsis (Fung et al., 2004). By contrast, in *Caenorhabditis elegans*, it was found that partial depletion of the central region proteins of SC attenuated genetic interference (Libuda et al., 2013). The partial loss of function of *ZEP1* mutants in rice showed similar results like that in *Caenorhabditis elegans* (Wang K. et al., 2015). Nonetheless, the exact role of SC in CO interference is still enigmatic because of the lack of null TF mutants. Recently, the *zyp1* null mutants in *Arabidopsis thaliana* were generated. Cytological and genetic analysis of the null mutants revealed that ZYP1 is required for CO interference (Capilla-Pérez et al., 2021; France et al., 2021). In this study, we found that genetic interference was eliminated in some detected genomic regions in our obtained rice *ZEP1* null mutants. The phenotype of *ZEP1* weak and null alleles in rice supports that the strength of genetic interference increased as the extent of synapsis increased.

Meiotic crossovers shuffle chromosomes to produce unique combinations of alleles that are transmitted to offspring (Mieulet et al., 2018). However, meiotic crossovers are tightly regulated by numerous of genes, typically one to three per chromosome (Fernandes et al., 2018). To combine favorable alleles into elite varieties, breeders and geneticists are exploring approaches to increase meiotic crossovers. Mutation of anti-crossover factors, such as FANCM, RECQ4, and FIGL1 in plants, induces a large increase in crossover frequency (Crismani et al., 2012; Girard et al., 2015; Séguéla-Arnaud et al., 2015; Mieulet et al., 2018). Reducing CO interference alters CO distribution, which also shows potential to enhance crop breeding efficiency (Taagen et al., 2020). However, it is very difficult to elevate genetic recombination frequency and eliminate genetic interference simultaneously in breeding process. Here, we used CRISPR/Cas9 to knock out *ZEP1*, encoding a component of the SC, in hybrid rice. The edited mutants concurrently exhibited disrupted CO interference and an elevated recombination frequency. Our study provides an effective approach to enrich genetic diversity and accelerate rice breeding. Of course, as the background play important roles in the formation of COs, more studies in different varieties are required to test its application potential in the future.

## Conclusion

In summary, we used CRISPR/Cas9 to completely knock out the *ZEP1* gene, encoding the central component of the SC, in hybrid rice. The mutants exhibited eliminated genetic interference and elevated genetic recombination frequency on chromosomes 1 and 8 in CY84 variety background. Our study of female gamete revealed the important role of the SC in mediating genetic interference and limiting COs. Very recently, null mutants of *ZYP1* in *Arabidopsis* were generated and a virtual absence of CO interference had been detected (Capilla-Pérez et al., 2021; France et al., 2021). Therefore, all of these studies support the long-term debating role of SC in mediating CO interference. Moreover, it is potential to break linkage drag and enrich genetic diversity by using *zep1* null mutants during rice breeding.

## Data Availability Statement

The datasets presented in this study can be found in online repositories. The names of the repository/repositories and accession number(s) can be found in the article/Supplementary Material.

## Author Contributions

KW managed the project. CL, YC, YH, GD, XW, TS, JL, and MW performed the experiments. YH, QL, and CL analyzed the data. CL and YH wrote the manuscript. KW and ZC revised the manuscript. All authors contributed to the article and approved the submitted version.

## Funding

This work was supported by the National Natural Science Foundation of China (32025028 and U20A2030), Central Public-interest Scientific Institution Basal Research Fund (Y2020XK17), the Agricultural Science and Technology Innovation Program (CAAS-ZDRW202001), and China National Rice Research Institute Key Research and Development Project (CNRRI-2020-01).

## Conflict of Interest

The authors declare that the research was conducted in the absence of any commercial or financial relationships that could be construed as a potential conflict of interest.

## Publisher’s Note

All claims expressed in this article are solely those of the authors and do not necessarily represent those of their affiliated organizations, or those of the publisher, the editors and the reviewers. Any product that may be evaluated in this article, or claim that may be made by its manufacturer, is not guaranteed or endorsed by the publisher.

## References

[ref1] Anderson L. K. Royer S. M. Page S. L. McKim K. S. Lai A. Lilly M. A.. (2005). Juxtaposition of C(2)M and the transverse filament protein C(3)G within the central region of *Drosophila* synaptonemal complex. Proc. Natl. Acad. Sci. U. S. A. 102, 4482–4487. doi: 10.1073/pnas.0500172102, PMID: 15767569 PMC555515

[ref2] Barakate A. Higgins J. D. Vivera S. Stephens J. Perry R. M. Ramsay L.. (2014). The synaptonemal complex protein ZYP1 is required for imposition of meiotic crossovers in barley. Plant Cell 26, 729–740. doi: 10.1105/tpc.113.121269, PMID: 24563202 PMC3967036

[ref3] Bishop D. K. Zickler D. (2004). Early decision: meiotic crossover interference prior to stable strand exchange and synapsis. Cell 117, 9–15. doi: 10.1016/S0092-8674(04)00297-1, PMID: 15066278

[ref4] Blary A. Jenczewski E. (2019). Manipulation of crossover frequency and distribution for plant breeding. Theor. Appl. Genet. 132, 575–592. doi: 10.1007/s00122-018-3240-1, PMID: 30483818 PMC6439139

[ref5] Capilla-Pérez L. Durand S. Hurel A. Lian Q. Chambon A. Taochy C.. (2021). The synaptonemal complex imposes crossover interference and heterochiasmy in *Arabidopsis*. Proc. Natl. Acad. Sci. U. S. A. 118:e2023613118. doi: 10.1073/pnas.2023613118, PMID: 33723072 PMC8000504

[ref6] Crismani W. Girard C. Froger N. Pradillo M. Santos J. L. Chelysheva L.. (2012). FANCM limits meiotic crossovers. Science 336, 1588–1590. doi: 10.1126/science.1220381, PMID: 22723424

[ref7] de Vries F. A. de Boer E. van den Bosch M. Baarends W. M. Ooms M. Yuan L.. (2005). Mouse *Sycp1* functions in synaptonemal complex assembly, meiotic recombination, and XY body formation. Genes Dev. 19, 1376–1389. doi: 10.1101/gad.329705, PMID: 15937223 PMC1142560

[ref400] de Boer E. Heyting C. (2006). The diverse roles of transverse filaments of synaptonemal complexes in meiosis. Chromosoma 115, 220–234. doi: 10.1007/s00412-006-0057-5, PMID: 16523321

[ref300] Dubois E. De Muyt A. Soyer J. L. Budin K. Legras M. Piolot T.. (2019). Building bridges to move recombination complexes. Natl. Acad. Sci. U. S. A. 116, 12400–12409. doi: 10.1073/pnas.1901237116, PMID: 31147459 PMC6589682

[ref8] Fernandes J. B. Séguéla-Arnaud M. Larchevêque C. Lloyd A. H. Mercier R. (2018). Unleashing meiotic crossovers in hybrid plants. Proc. Natl. Acad. Sci. U. S. A. 115, 2431–2436. doi: 10.1073/pnas.1713078114, PMID: 29183972 PMC5877974

[ref9] France M. G. Enderle J. Röhrig S. Puchta H. Franklin F. Higgins J. D. (2021). ZYP1 is required for obligate cross-over formation and cross-over interference in *Arabidopsis*. Proc. Natl. Acad. Sci. U. S. A. 118:e2021671118. doi: 10.1073/pnas.2021671118, PMID: 33782125 PMC8040812

[ref10] Fraune J. Brochier-Armanet C. Alsheimer M. Volff J. N. Schücker K. Benavente R. (2016). Evolutionary history of the mammalian synaptonemal complex. Chromosoma 125, 355–360. doi: 10.1007/s00412-016-0583-8, PMID: 26968413

[ref11] Fung J. C. Rockmill B. Odell M. Roeder G. S. (2004). Imposition of crossover interference through the nonrandom distribution of synapsis initiation complexes. Cell 116, 795–802. doi: 10.1016/S0092-8674(04)00249-1, PMID: 15035982

[ref12] Gao J. Colaiácovo M. P. (2018). Zipping and unzipping: protein modifications regulating synaptonemal complex dynamics. Trends Genet. 34, 232–245. doi: 10.1016/j.tig.2017.12.001, PMID: 29290403 PMC5834363

[ref13] Girard C. Chelysheva L. Choinard S. Froger N. Macaisne N. Lemhemdi A.. (2015). AAA-ATPase FIDGETIN-LIKE 1 and helicase FANCM antagonize meiotic crossovers by distinct mechanisms. PLoS Genet. 11:e1005369. doi: 10.1371/journal.pgen.1005369, PMID: 26161528 PMC4498898

[ref14] Golubovskaya I. N. Wang C. J. Timofejeva L. Cande W. Z. (2011). Maize meiotic mutants with improper or non-homologous synapsis due to problems in pairing or synaptonemal complex formation. J. Exp. Bot. 62, 1533–1544. doi: 10.1093/jxb/erq292, PMID: 20926553 PMC3107535

[ref15] Higgins J. D. Sanchez-Moran E. Armstrong S. J. Jones G. H. Franklin F. C. (2005). The *Arabidopsis* synaptonemal complex protein ZYP1 is required for chromosome synapsis and normal fidelity of crossing over. Genes Dev. 19, 2488–2500. doi: 10.1101/gad.354705, PMID: 16230536 PMC1257403

[ref16] Khoo K. H. Able A. J. Able J. A. (2012). The isolation and characterisation of the wheat molecular ZIPper I homologue, *Ta*ZYP1. BMC. Res. Notes 5:106. doi: 10.1186/1756-0500-5-106, PMID: 22340255 PMC3305362

[ref18] Libuda D. E. Uzawa S. Meyer B. J. Villeneuve A. M. (2013). Meiotic chromosome structures constrain and respond to designation of crossover sites. Nature 502, 703–706. doi: 10.1038/nature12577, PMID: 24107990 PMC3920622

[ref19] Liu C. L. Gao Z. Y. Shang L. G. Yang C. H. Ruan B. P. Zeng D. L.. (2020). Natural variation in the promoter of *OsHMA3* contributes to differential grain cadmium accumulation between *Indica* and *japonica* rice. J. Integr. Plant Biol. 62, 314–329. doi: 10.1111/jipb.12794, PMID: 30791211

[ref20] Liu Q. Wang C. Jiao X. Zhang H. Song L. Li Y.. (2019). Hi-TOM: a platform for high-throughput tracking of mutations induced by CRISPR/Cas systems. Sci. China Life Sci. 62, 1–7. doi: 10.1007/s11427-018-9402-9, PMID: 30446870

[ref22] MacQueen A. J. Colaiácovo M. P. McDonald K. Villeneuve A. M. (2002). Synapsis-dependent and -independent mechanisms stabilize homolog pairing during meiotic prophase in *C. elegans*. Genes Dev. 16, 2428–2442. doi: 10.1101/gad.1011602, PMID: 12231631 PMC187442

[ref23] Mieulet D. Aubert G. Bres C. Klein A. Droc G. Vieille E.. (2018). Unleashing meiotic crossovers in crops. Nat. Plants 4, 1010–1016. doi: 10.1038/s41477-018-0311-x, PMID: 30478361

[ref24] Moose S. P. Mumm R. H. (2008). Molecular plant breeding as the foundation for 21st century crop improvement. Plant Physiol. 147, 969–977. doi: 10.1104/pp.108.118232, PMID: 18612074 PMC2442525

[ref25] Moses M. J. (1969). Structure and function of the synaptonemal complex. Genetics 61, 41–51. PMID: 5345399

[ref26] Otto S. P. Payseur B. A. (2019). Crossover interference: shedding light on the evolution of recombination. Annu. Rev. Genet. 53, 19–44. doi: 10.1146/annurev-genet-040119-093957, PMID: 31430178 PMC8715713

[ref27] Page S. L. Hawley R. S. (2001). *c*(3)*G* encodes a *Drosophila* synaptonemal complex protein. Genes Dev. 15, 3130–3143. doi: 10.1101/gad.935001, PMID: 11731477 PMC312841

[ref29] Schild-Prüfert K. Saito T. T. Smolikov S. Gu Y. Hincapie M. Hill D. E.. (2011). Organization of the synaptonemal complex during meiosis in *Caenorhabditis elegans*. Genetics 189, 411–421. doi: 10.1534/genetics.111.132431, PMID: 21840865 PMC3189812

[ref30] Séguéla-Arnaud M. Crismani W. Larchevêque C. Mazel J. Froger N. Choinard S.. (2015). Multiple mechanisms limit meiotic crossovers: TOP3α and two BLM homologs antagonize crossovers in parallel to FANCM. Proc. Natl. Acad. Sci. U. S. A. 112, 4713–4718. doi: 10.1073/pnas.1423107112, PMID: 25825745 PMC4403193

[ref31] Storlazzi A. Xu L. Schwacha A. Kleckner N. (1996). Synaptonemal complex (SC) component Zip1 plays a role in meiotic recombination independent of SC polymerization along the chromosomes. Proc. Natl. Acad. Sci. U. S. A. 93, 9043–9048. doi: 10.1073/pnas.93.17.9043, PMID: 8799151 PMC38592

[ref32] Sun L. Wang J. Sang M. Jiang L. Zhao B. Cheng T.. (2017). Landscaping crossover interference across a genome. Trends Plant Sci. 22, 894–907. doi: 10.1016/j.tplants.2017.06.008, PMID: 28822625

[ref33] Sym M. Engebrecht J. A. Roeder G. S. (1993). ZIP1 is a synaptonemal complex protein required for meiotic chromosome synapsis. Cell 72, 365–378. doi: 10.1016/0092-8674(93)90114-6, PMID: 7916652

[ref34] Taagen E. Bogdanove A. J. Sorrells M. E. (2020). Counting on crossovers: controlled recombination for plant breeding. Trends Plant Sci. 25, 455–465. doi: 10.1016/j.tplants.2019.12.017, PMID: 31959421

[ref36] Wang C. Liu Q. Shen Y. Hua Y. Wang J. Lin J.. (2019). Clonal seeds from hybrid rice by simultaneous genome engineering of meiosis and fertilization genes. Nat. Biotechnol. 37, 283–286. doi: 10.1038/s41587-018-0003-0, PMID: 30610223

[ref37] Wang C. Shen L. Fu Y. Yan C. Wang K. (2015). A simple CRISPR/Cas9 system for multiplex genome editing in rice. J. Genet. Genomics 42, 703–706. doi: 10.1016/j.jgg.2015.09.011, PMID: 26743988

[ref500] Wang K. Tang D. Wang M. Lu J. Yu H. Liu J.. (2009). MER3 is required for normal meiotic crossover formation, but not for presynaptic alignment in rice. J Cell Sci. 122, 2055–2063. doi: 10.1242/jcs.049080, PMID: 19470578

[ref38] Wang K. Wang C. Liu Q. Liu W. Fu Y. (2015). Increasing the genetic recombination frequency by partial loss of function of the synaptonemal complex in rice. Mol. Plant 8, 1295–1298. doi: 10.1016/j.molp.2015.04.011, PMID: 25936677

[ref39] Wang M. Wang K. Tang D. Wei C. Li M. Shen Y.. (2010). The central element protein ZEP1 of the synaptonemal complex regulates the number of crossovers during meiosis in rice. Plant Cell 22, 417–430. doi: 10.1105/tpc.109.070789, PMID: 20154151 PMC2845403

[ref40] Zhang L. Espagne E. de Muyt A. Zickler D. Kleckner N. E. (2014). Interference-mediated synaptonemal complex formation with embedded crossover designation. Proc. Natl. Acad. Sci. U. S. A. 111, E5059–E5068. doi: 10.1073/pnas.1416411111, PMID: 25380597 PMC4250137

[ref41] Zhang L. Tao J. Wang S. Chong K. Wang T. (2006). The rice OsRad21-4, an orthologue of yeast Rec8 protein, is required for efficient meiosis. Plant Mol. Biol. 60, 533–534. doi: 10.1007/s11103-005-4922-z, PMID: 16525890

