# Supplementary figures and images for: Concurrent Disruption of Genetic Interference and Increase of Genetic Recombination Frequency in Hybrid Rice Using CRISPR/Cas9
Source: Front Plant Sci. 2021 Oct 1;12:757152. doi: 10.3389/fpls.2021.757152 (PMC8523357; doi:10.3389/fpls.2021.757152)

## Slide 1
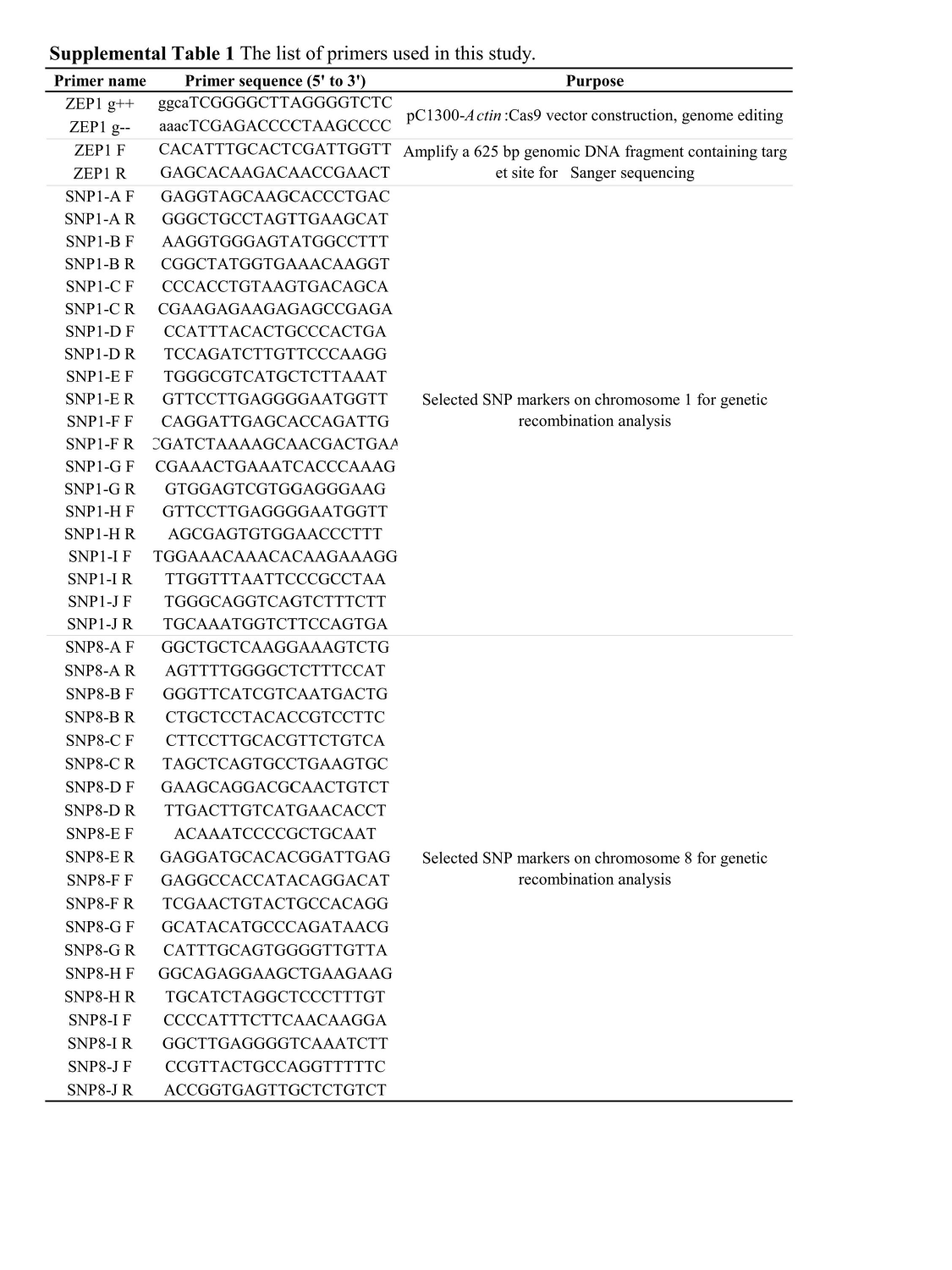

## Slide 2
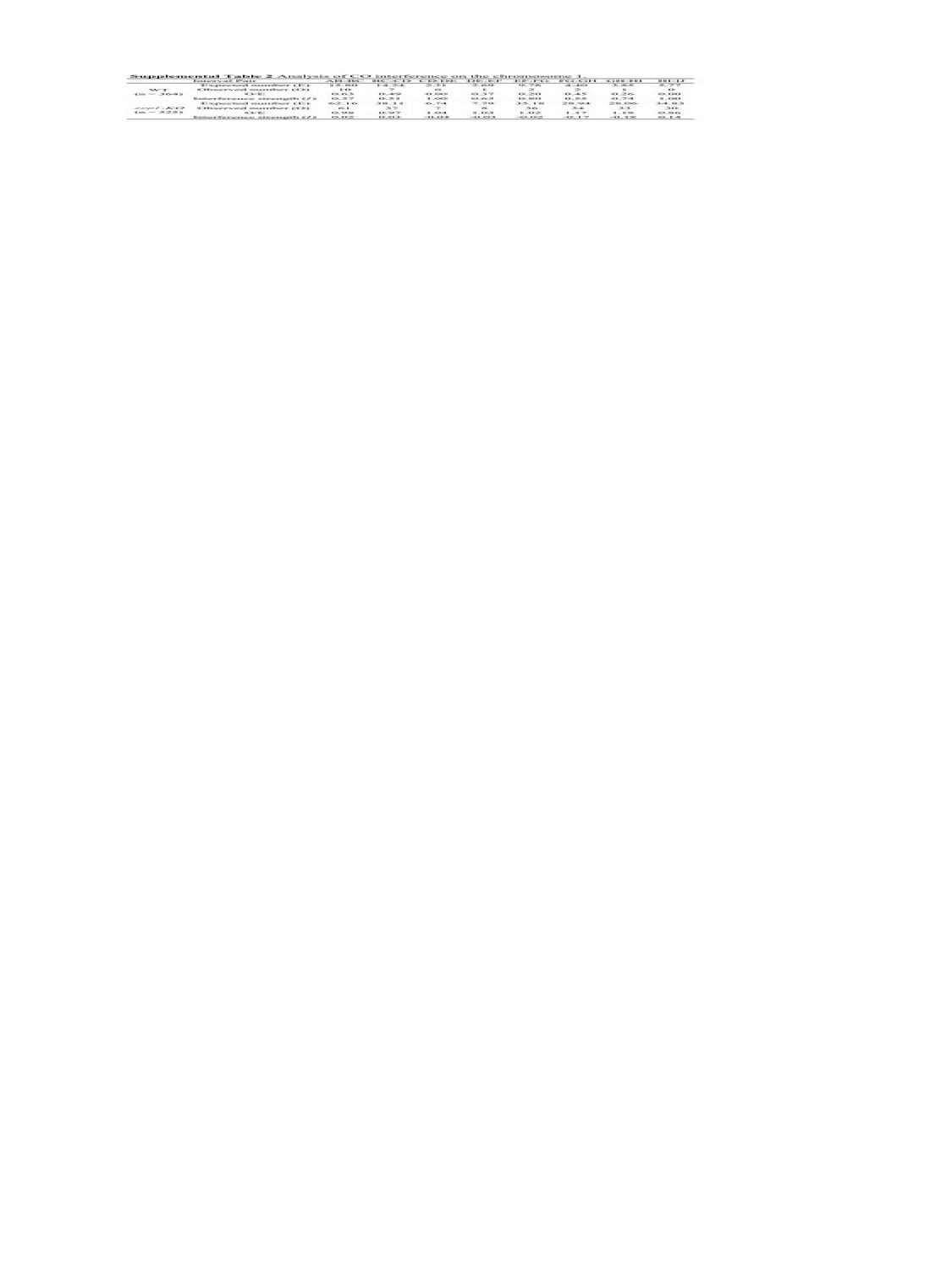

## Slide 3
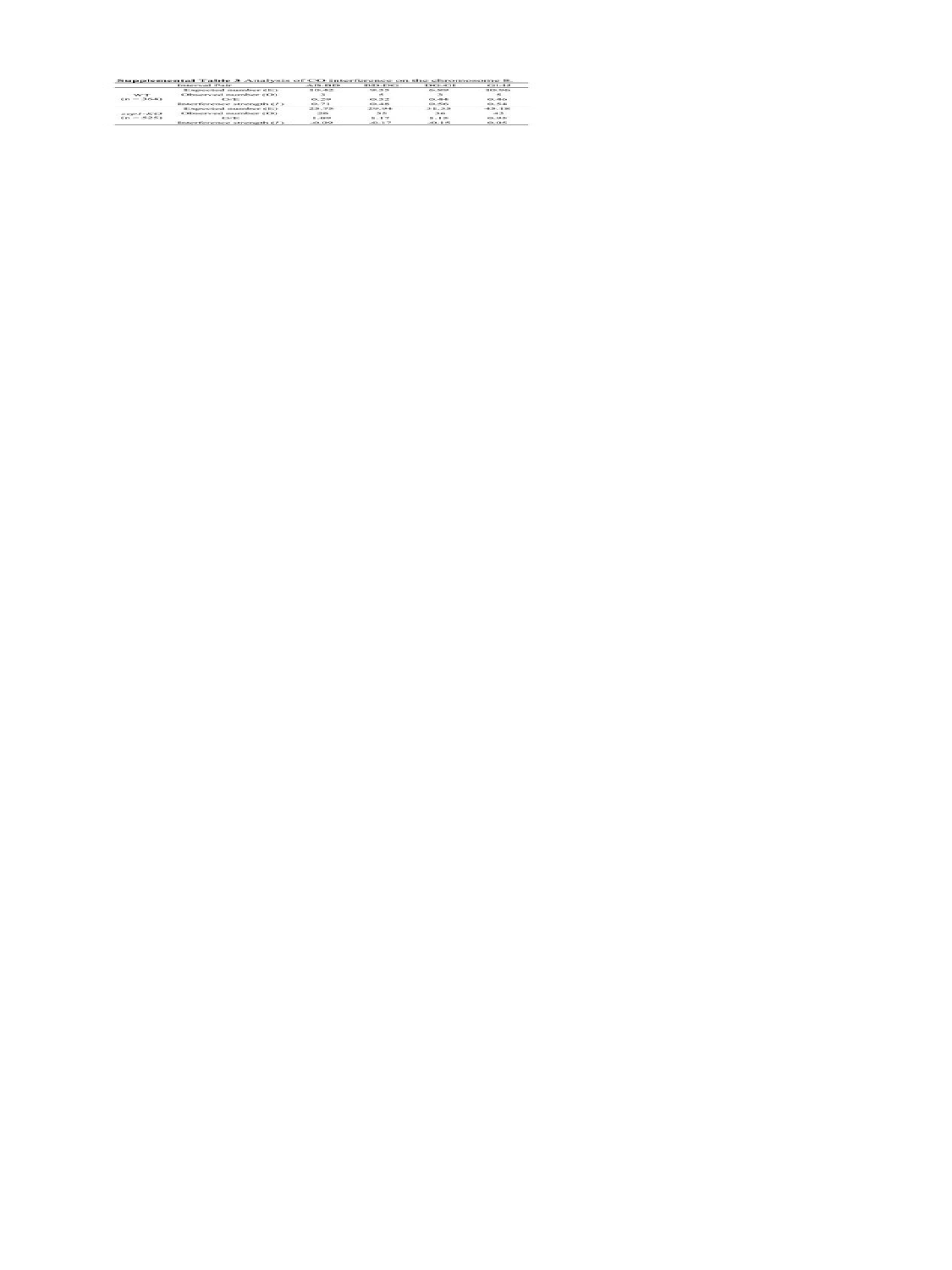

Supplement: Supplementary file 1 [file Presentation_1.PPT]
